# Assessing the representativeness of physician and patient respondents to a primary care survey using administrative data

**DOI:** 10.1186/s12875-018-0767-9

**Published:** 2018-05-30

**Authors:** Allanah Li, Shawna Cronin, Yu Qing Bai, Kevin Walker, Mehdi Ammi, William Hogg, Sabrina T. Wong, Walter P. Wodchis

**Affiliations:** 1 Institute of Health Policy, Management and Evaluation, University of Toronto, Toronto, Canada; 2 Department of Family & Community Medicine, University of Toronto, Toronto, Canada; 3 Institute for Clinical Evaluative Sciences, Toronto, Canada; 4 School of Public Policy & Administration, Carleton University, Ottawa, Canada; 5 Department of Family Medicine, University of Ottawa, Ottawa, Canada; 6 Bruyere Research Institute, Ottawa, Canada; 7 School of Nursing, University of British Columbia, Vancouver, Canada; 8 Centre for Health Services and Policy Research, University of British Columbia, Vancouver, Canada; 9 Toronto Rehabilitation Institute, Toronto, Canada

**Keywords:** Primary care, Survey bias, Canada, Representativeness

## Abstract

**Background:**

QUALICOPC is an international survey of primary care performance. QUALICOPC data have been used in several studies, yet the representativeness of the Canadian QUALICOPC survey is unknown, potentially limiting the generalizability of findings. This study examined the representativeness of QUALICOPC physician and patient respondents in Ontario using health administrative data.

**Methods:**

This representativeness study linked QUALICOPC physician and patient respondents in Ontario to health administrative databases at the Institute for Clinical Evaluative Sciences. Physician respondents were compared to other physicians in their practice group and all Ontario primary care physicians on demographic and practice characteristics. Patient respondents were compared to other patients rostered to their primary care physicians, patients rostered to their physicians’ practice groups, and a random sample of Ontario residents on sociodemographic characteristics, morbidity, and health care utilization. Standardized differences were calculated to compare the distribution of characteristics across cohorts.

**Results:**

QUALICOPC physician respondents included a higher proportion of younger, female physicians and Canadian medical graduates compared to other Ontario primary care physicians. A higher proportion of physician respondents practiced in Family Health Team models, compared to the provincial proportion for primary care physicians. QUALICOPC patient respondents were more likely to be older and female, with significantly higher levels of morbidity and health care utilization, compared with the other patient groups examined. However, when looking at the QUALICOPC physicians’ whole rosters, rather than just the patient survey respondents, the practice profiles were similar to those of the other physicians in their practice groups and Ontario patients in general.

**Conclusions:**

Comparisons revealed some differences in responding physicians’ demographic and practice characteristics, as well as differences in responding patients’ characteristics compared to the other patient groups tested, which may have resulted from the visit-based sampling strategy. Ontario QUALICOPC physicians had similar practice profiles as compared to non-participating physicians, providing some evidence that the participating practices are representative of other non-participating practices, and patients selected by visit-based sampling may also be representative of visiting patients in other practices. Those using QUALICOPC data should understand this limited representativeness when generalizing results, and consider the potential for bias in their analyses.

**Electronic supplementary material:**

The online version of this article (10.1186/s12875-018-0767-9) contains supplementary material, which is available to authorized users.

## Background

Ongoing primary care reform in Canada and around the world has spurred a need for comprehensive and meaningful measurement of primary care performance [1]. This is the case for primary care in the Canadian province of Ontario, where, despite being publicly funded and central to the health care system, there is a paucity of high quality data on primary care performance [2].

Surveys are an important source of information in health services research, policy, and planning. However, physician surveys often have low response rates, which may introduce concern about their validity and representativeness [3]. While response rate is sometimes used as a marker of survey quality, Halbesleben and Whitman advocate for looking beyond response rates when assessing the quality of survey data [4]. They recommend examining nonresponse bias, which occurs when there is a systematic difference between those who do and do not respond to a survey [4]. One common method of assessing nonresponse bias in physician surveys is to compare respondents and nonrespondents, on the basis of demographic and practice characteristics [5–13]. These comparisons have identified differences in responding physicians compared to nonrespondents, including differences in age, gender, and years of schooling [7, 8].

There is limited research exploring nonresponse bias in primary care patient surveys. Some studies have found that patient surveys have potential for biased results due to method of survey administration [14–17]. One study identified differences in gender, income, and age among patients who responded to a survey in the waiting room compared to those who responded by e-mail [16]. Another found telephone survey response rates differed by patients’ age, tobacco use, and comorbidity scores [18].

The Quality and Costs of Primary Care study (QUALICOPC) is a multi-national study on primary care performance investigating quality, equity, and costs of primary care in 34 countries worldwide [2, 19]. Details of the design and administration of the Canadian QUALICOPC can be found elsewhere [2, 20]. Briefly, research teams from each province in Canada collected data for the QUALICOPC study in 2013 and early 2014. The QUALICOPC study included practice, physician, and patient surveys. In Ontario, a random sample of physicians was not possible as researchers did not have access to a list of eligible physicians. Instead, emails were sent by the Ontario College of Family Physicians inviting eligible physicians working in general/family practice to register for the study. The family physician survey was completed by the participating physician and the practice survey was completed by either the participating physician or administrative staff. For the patient surveys, physicians were instructed to choose a day they felt represented their regular practice population, and surveys were distributed by practice staff to consecutive consenting adult patients visiting the practice that day.

While QUALICOPC represents the largest study on quality, organization, and patient values and experiences in primary care in Canada, limited resources were available for provider recruitment and response rates for physicians across the country were generally low, ranging from 2% in British Columbia to 21% in Nova Scotia [2]. In Ontario, Canada’s most populous province, the response rate was 3% [2]. With low physician response rates from a self-selected sample, and the corresponding patient sample consisting of consecutive visit-based sampling, it is unknown to what extent the QUALICOPC physician and patient respondents can be generalized to the province. If the respondents are not representative of the province, then are they representative of meaningful subgroups, such as the other physicians and patients in the same practice? These comparisons can help determine to what extent physician, patient, or practice-level inferences are valid.

The objective of the current study was to examine the representativeness of QUALICOPC physician and patient respondents in Ontario by answering the following questions:

1. To what extent are the QUALICOPC physician respondents representative of i) the physicians in their practice group, and ii) other primary care physicians in Ontario?
2. To what extent are the QUALICOPC patient respondents representative of i) the patients in their physicians’ rosters, ii) the patients in their physicians’ groups’ rosters, and iii) the general Ontario population?

## Methods

### Measures and data sources

This representativeness study linked a database of QUALICOPC survey respondents to health administrative databases held at the Institute for Clinical Evaluative Sciences (ICES). Data holdings at ICES include a number of databases with information on providers and patients, such as physician billings, hospital inpatient and emergency room care, and census data [17, 21]. Universal health insurance in Canada means that the databases capture the whole population. Physician databases and public data have been used to examine survey representativeness [7, 22], while census data has been used to examine representativeness of an EMR database [23]. Administrative data were captured from January 1, 2013 to December 31, 2013, corresponding to the period of data collection in Ontario. For the physician cohorts, 185 physicians completed QUALICOPC surveys in Ontario, and we were successful in linking 175 (95%) of these respondents with administrative data using unique encrypted physician billing numbers. Using group number, a variable identifying groups of physicians practicing together, we then identified the other primary care physicians belonging to the same practice groups as the survey respondents. We also identified the remaining primary care physicians in Ontario.

Of the 1698 patients who completed the QUALICOPC patient experience survey, 1225 (72%) consented to linkage to health administrative databases using their health card numbers. We then identified the other patients in their physicians’ rosters, as well as the other patients in their physicians’ practice groups’ rosters. We included patients who were formally or virtually rostered to the primary care physicians; formally rostered patients had signed an enrollment form while virtually rostered patients were those who saw a particular physician for the majority of their visits over the previous year. Finally, in order to construct a provincially representative sample of patients for comparison, we also determined a 10% simple random sample of all patients in Ontario aged 18 and older with a valid health card number.

The physician cohorts were compared on demographic characteristics, including sex, age, years since graduation, and whether they were Canadian medical graduates. They were also compared on type of primary care practice model they were practicing in, and roster size. The patient cohorts were compared on sociodemographic characteristics, including sex, age, material deprivation, and rurality, as well as morbidity and health care utilization, including primary care visits, emergency department visits, and acute care hospitalizations. These variables are commonly found to vary among respondents and nonrespondents in other studies. Furthermore, these variables may be related to primary care performance and patient experience, and are thus important to examine in the context of a primary care performance survey [16, 18].

Demographic and practice information for physicians was derived from the ICES Physician Database (IPDB) and Client Agency Program Enrolment (CAPE) tables. Primary care models were also derived from CAPE, and classified according to type of practice (solo vs. group) and remuneration: solo physicians (including enhanced fee for service and fee for service), group enhanced fee for service (i.e. Family Health Group), group capitated (i.e. Family Health Organization), and group capitated with an allied health team (i.e. Family Health Team). Family Health Network and Other group models were not included in the analysis as they each had fewer than 6 physician respondents in the QUALICOPC. See Additional file 1 for a summary of primary care models in Ontario. Since solo physicians, by definition, do not belong to a practice group, they were only compared to the other Ontario primary care physicians.

Demographic information for patients, including age, sex, and postal code, was derived from the Registered Person’s Database (RPDB). Health care utilization was derived from the National Ambulatory Care Reporting System (NACRS), the Canadian Institute of Health Information Discharge Abstract Database (CIHI-DAD), and the Ontario Health Insurance Plan (OHIP).

Rurality of the patients was measured using the Rurality Index of Ontario (RIO), a scale which assigns a number score between 0 and 100 using postal codes and an algorithm which takes into account population density and travel times to referral centres. RIO scores of 0–9 were considered urban, 10–39 as suburban, and 40 or greater as rural [24].

Material deprivation of the patients was measured using the Canadian Marginalization Index, which is derived geographically from census data and includes measures such as proportion of the population without a high school diploma, proportion of households living in dwellings that are in need of major repair, and proportion of the population above the age of 15 who are unemployed [25].

To account for the morbidity burden of the patients, resource utilization bands (RUBs) were used. RUBs are part of the Johns Hopkins Adjusted Clinical Groups Casemix system and are derived from hospitalization and primary care visit records. RUBs range from 0 (non-users of the health care system) to 5 (very high users) [26]. The prevalence of five specific chronic diseases was determined using validated cohort databases at ICES: asthma, chronic obstructive pulmonary disease (COPD), congestive heart failure (CHF), hypertension, and diabetes.

### Analysis

The standardized difference, also known as effect size, was calculated to compare the means and proportions of variables across the physician and patient comparison groups. The standardized difference was selected as it is not as sensitive to large sample sizes, such as those in our study, as traditional significance tests and it also provides information about the relative magnitude of differences between groups [27]. Consistent with Cohen (1988, as described in [28]), we considered a standardized difference of 0.2 to indicate a small, but meaningful difference between groups. All analyses were conducted in SAS version 9.4.

## Results

### Physician respondents

Data from 175 physician QUALICOPC respondents were compared to 2507 physicians in the same practice groups, and 9758 Ontario primary care physicians (Table 1). Physician respondents were, on average, younger, had fewer years of experience, and consisted of a higher proportion of female physicians compared to the other physicians in their practice groups, though these standardized differences were mostly below 0.2, with larger differences when comparing respondents to the Ontario primary care physicians. Survey respondents included a smaller proportion of physicians who attended medical school abroad, with 19.4% international medical graduates compared to 27.7% in Ontario. While roster sizes were comparable, survey respondents consisted of fewer solo physicians and more who practiced in Family Health Teams as compared to the Ontario average.

**Table 1:** QUALICOPC physician respondents compared with physicians in their practice groups and Ontario primary care physicians

	Group 1: QUALICOPC physician respondents	Group 2: QUALICOPC physicians’ practice groups	Group 3: Ontario primary care physicians	Standardized difference^a^	
				Group 2 vs. 1	Group 3 vs. 1
	*N* = 175	*N* = 2507	*N* = 9758		
Sex, N (%)					
Female	98 (56.0)	1177 (47.0)	4110 (42.1)	0.18	*0.28*
Male	77 (44.0)	1330 (53.0)	5642 (57.8)	0.18	*0.28*
Age, mean (SD)	49 (10)	51 (11)	51 (12)	0.19	*0.20*
Years in practice, mean (SD)	23 (11)	25 (12)	25 (13)	*0.20*	*0.21*
Canadian medical graduate, N (%)					
Yes	141 (80.6)	1878 (74.9)	7054 (72.3)	0.14	*0.20*
No	34 (19.4)	629 (25.1)	2698 (27.7)	0.14	*0.20*
Roster size, mean (SD)	1257 (582)	1126 (786)	1120 (1045)	0.19	0.16
Primary care model^b^, N(%)					
Solo physicians	12 (6.9)	0	3711 (38.0)	-	*0.81*
FHG	44 (25.1)	1117 (44.6)	2415 (24.8)	*0.42*	0.01
FHN	< 6	27 (1.1)	202 (2.1)	-	-
FHO	38 (21.7)	401 (16.0)	1765 (18.1)	0.15	0.09
FHT	73 (41.7)	923 (36.8)	1594 (16.3)	0.10	*0.58*
Other group	< 6	39 (1.6)	71 (0.7)	-	-

*SD* standard deviation, *FHG* Family Health Group, *FHN* Family Health Network, *FHO* Family Health Organization, *FHT* Family Health Team

^a^Clarify that standardized differences >=0.2 are considered a meaningful difference and are highlighted in italics

^b^Primary care models are classified according to type of practice model and remuneration: solo physicians (including enhanced fee for service and fee for service), group enhanced fee for service (i.e. Family Health Group), group capitated (i.e. Family Health Organization), and group capitated with an allied health team (i.e. Family Health Team). Family Health Network and Other group models were not included in the analysis as they each had fewer than 6 physician respondents in the QUALICOPC

### Patient respondents

In total, 1225 patient respondents to the QUALICOPC study were compared to 158,537 patients within participating physicians’ rosters, 2,270,380 patients rostered to the participating physicians’ practice groups, and 831,056 patients representing a 10% simple random sample of Ontarians aged 18 years and older (Table 2).

**Table 2:** QUALICOPC patient respondents compared with patients in their physicians’ rosters, practice groups’ rosters, and Ontario

	Group 1: QUALICOPC patient respondents *N* = 1225	Group 2: QUALICOPC physicians’ rosters *N* = 158,537	Group 3: QUALICOPC physicians’ practice groups’ rosters *N* = 2,270,380	Group 4: Ontario population, 10% simple random sample *N* = 831,056	Standardized difference^a^				
					Group 2 vs. 1	Group 3 vs. 1	Group 4 vs. 1	Group 3 vs. 2	Group 4 vs. 2
Sex, N (%)									
Female	782 (63.8)	88,682 (55.9)	1244,3224 (54.8)	420,085 (50.5)	0.16	0.18	*0.27*	0.02	0.11
Male	443 (36.2)	69,855 (44.1)	1,026,056 (45.2)	410,971 (49.5)	0.16	0.18	*0.27*	0.02	0.11
Age, N (%)									
18–44	423 (34.5)	69,786 (44.0)	987,543 (43.5)	385,125 (46.3)	*0.20*	0.18	*0.24*	0.01	0.05
45–64	492 (40.2)	57,281 (36.1)	829,440 (36.5)	291,929 (35.1)	0.08	0.07	0.10	0.01	0.02
65+	310 (25.3)	31,470 (19.9)	453,397 (20.0)	154,002 (18.5)	0.13	0.13	0.16	0.00	0.03
Material deprivation quintile, N (%)									
1 (least deprived)	303 (25.1)	39,917 (25.7)	561,391 (25.1)	187,235 (22.9)	0.01	0.00	0.05	0.01	0.06
2	266 (22.0)	33,362 (21.4)	471,281 (21.1)	162,865 (20.0)	0.01	0.02	0.05	0.01	0.04
3	208 (17.2)	28,781 (18.5)	421,191 (18.9)	157,343 (19.3)	0.03	0.04	0.05	0.01	0.02
4	219 (18.1)	27,392 (17.6)	405,012 (18.1)	149,790 (18.4)	0.01	0.00	0.01	0.01	0.02
5 (most deprived)	211 (17.5)	26,084 (16.8)	373,716 (16.7)	158,614 (19.4)	0.02	0.02	0.05	0.00	0.07
Rurality Index of Ontario, N (%)									
< 10 (least rural)	795 (64.9)	104,620 (66.0)	1,688,247 (74.4)	608,395 (73.2)	0.02	*0.21*	0.18	0.18	0.16
10–40	316 (25.8)	39,638 (25.0)	463,532 (20.4)	155,884 (18.8)	0.02	0.13	0.17	0.11	0.15
40+ (most rural)	114 (9.3)	14,279 (9.0)	118,601 (5.2)	66,777 (8.0)	0.01	0.16	0.05	0.15	0.03
Resource utilization bands, N (%)									
0 (non-user)	22 (1.8)	8856 (5.6)	133,031 (5.9)	92,008 (11.1)	*0.20*	*0.21*	*0.38*	0.01	*0.20*
1 (healthy user)	35 (2.9)	8746 (5.5)	120,487 (5.3)	49,519 (6)	0.13	0.12	0.15	0.01	0.02
2 (low morbidity)	87 (7.1)	26,594 (16.8)	376,574 (16.6)	141,200 (17)	*0.30*	*0.30*	*0.31*	0.01	0.01
3 (moderate morbidity)	662 (54.0)	81,632 (51.5)	1,185,228 (52.2)	397,248 (47.8)	0.05	0.04	0.13	0.01	0.07
4 (high morbidity)	292 (23.8)	24,256 (15.3)	339,211 (14.9)	111,158 (13.4)	*0.22*	*0.23*	*0.27*	0.01	0.05
5 (very high morbidity)	127 (10.4)	8453 (5.3)	115,849 (5.1)	39,923 (4.8)	0.19	*0.20*	*0.21*	0.01	0.02
Chronic disease, N (%)									
Asthma	255 (20.8)	22,792 (14.4)	329,256 (14.5)	112,173 (13.5)	0.17	0.17	*0.20*	0.00	0.03
COPD	56 (4.6)	4285 (2.7)	61,555 (2.7)	21,446 (2.6)	0.10	0.10	0.11	0.00	0.01
CHF	44 (3.6)	3523 (2.2)	51,127 (2.3)	18,228 (2.2)	0.08	0.08	0.08	0.00	0.00
Hypertension	446 (36.4)	43,269 (27.3)	640,891 (28.2)	213,398 (25.7)	*0.20*	0.18	*0.23*	0.02	0.04
Diabetes	206 (16.8)	18,653 (11.8)	277,755 (12.2)	93,806 (11.3)	0.14	0.13	0.16	0.01	0.01
Healthcare visits in the last year, mean (SD)									
Primary care	5.83 (6.24)	3.46 (4.08)	3.69 (4.32)	3.33 (4.38)	*0.45*	*0.40*	*0.46*	0.05	0.03
Emergency department	0.58 (1.23)	0.44 (1.23)	0.40 (1.15)	0.42 (1.25)	0.12	0.16	0.13	0.03	0.01
Acute care	0.12 (0.47)	0.08 (0.36)	0.08 (0.36)	0.07 (0.36)	0.10	0.11	0.12	0.01	0.02

*SD* standard deviation, *COPD* chronic obstructive pulmonary disease, *CHF* congestive heart failure

^a^Clarify that standardized differences >=0.2 are considered a meaningful difference and are highlighted in italics

Patient survey respondents consisted of a greater proportion of female patients compared to the population of Ontario. Survey respondents also included a lower proportion of patients between the ages of 18 and 44, compared to their physicians’ rosters and the population of Ontario. Patients surveyed did not differ from the other patient populations in terms of material deprivation, with 17% of respondents living in areas with high deprivation compared to 17% for the physicians’ rosters and 19% for the province, and all standardized differences less than 0.2.

QUALICOPC survey respondents had more comorbidities as measured by RUBs than any of the other patient populations. Survey respondents had a lower proportion of “low morbidity,” and higher proportions of “high morbidity” and “very high morbidity” patients than comparator groups, with survey respondents including 24% “high morbidity,” compared to 15% in their physicians’ and practice groups’ rosters. Survey respondents had some differences in terms of specific chronic conditions, demonstrating higher prevalence of asthma and hypertension compared to the province. However, there were not meaningful differences in COPD, CHF, or diabetes across the comparison groups.

Survey respondents were also more frequent users of the health care system, with an average of 5.83 primary care visits per year, compared to an average of 3.46 visits for the other patients in their physicians’ rosters, 3.69 in the practice groups’ rosters, and the provincial average of 3.33. Emergency department visits and number of hospitalizations demonstrated a similar trend, but standardized differences were less than 0.2.

Notably, when we looked at the QUALICOPC physicians’ whole rosters, rather than just the patient survey respondents, the patient characteristics were very similar to those of the other physicians in their practice groups and Ontario patients in general (group 2 vs. group 3 and group 4 in Table 2). The only meaningful difference, according to our threshold, was when looking at morbidity using RUBs, where there was a higher proportion of “non-users” in the province compared to the QUALICOPC physicians’ rosters.

## Discussion

This is the first study to examine the representativeness of the QUALICOPC study within Canada. While other studies have explored the representativeness of QUALICOPC physician respondents internationally, this is also the first study to assess representativeness of both the physician and patient respondents using comprehensive administrative databases. In one QUALICOPC study from Switzerland, primary care physicians were randomly selected from a database by mail to participate in the survey, with another set of randomly selected physicians as the comparison. Physician survey respondents were found to be similar to their comparators in terms of age, sex and practice location [29]. Another QUALICOPC study from Australia assessed nonresponse bias by contacting nonresponders by telephone; researchers concluded that the gender split of physicians was similar, but younger primary care physicians were underrepresented in the survey sample [10]. These differences are likely due to the variability in sampling and recruitment used in the different iterations of the QUALICOPC study internationally. The findings in our study may relate to the fact that in Ontario the physician respondents were self-selected (i.e. invitations were sent to all physicians), whereas in the Swiss and Australian contexts they were recruited by random sampling.

Physician survey respondents were younger on average than nonrespondent physicians, which is consistent with literature exploring nonresponse bias in primary care surveys for physicians [5–8, 13]. A minority of studies have concluded the opposite; however, these studies used a random sampling strategy and were conducted within different geographical contexts [10, 11]. It has been suggested that differences in how physicians are trained may help to explain why age is associated with survey responses. If this is indeed the case, it may be that Ontario physicians that have graduated more recently have more interest in participating in research and primary care performance measurement than their more experienced counterparts.

We identified that physician survey respondents included more physicians who worked in Family Health Teams, rather than solo practice, a conclusion that is consistent with literature on this topic. It has been suggested that one of the reasons for this is that physicians working in groups have more time to devote to non-patient care, and may be more likely to complete a survey [5, 7, 11, 12]. The opportunity cost of answering a survey would be higher for physicians paid by fee for service compared to those paid by capitation, such as those in a Family Health Team. Our finding that a higher proportion of physician respondents were local rather than international medical graduates is also consistent with the literature [13].

The patient respondents were recruited by consecutive visit-based sampling in primary care, which means they were patients with access to primary care who are more likely to need or use these services. Consistent with our findings regarding the characteristics of patient respondents, another Ontario primary care study also found that patients recruited by consecutive sampling in the waiting room sampled a population that was older, sicker, and more likely to be female compared to the rest of the practice population [17]. Similarly, an American study of visit-based sampling in Veterans Affairs primary care firms found that patients sampled were older, had more visits, and were in poorer health compared to the general patient population [30].

In addition, the sampling method used in the Ontario QUALICOPC study involved first recruiting one physician from each practice, followed by recruiting the patients of the responding physicians, meaning that patient respondents were dependent on which physicians responded to the survey. Another study that explored this recruitment strategy found no difference between patients whose physicians participated and those whose physicians did not participate [31]. In our study, we also found that the respondent physicians’ patient rosters were similar to those of their practice groups and the Ontario population, although there were differences between participating and non-participating physicians’ demographic and practice characteristics. Thus differences observed between the patient respondents and the general population are more likely due to the visit-based patient sampling methods than differences in the patient rosters of responding physicians.

Given that the profile of QUALICOPC physicians’ whole rosters were similar to their practice groups’ and the province, there is some evidence that the participating practices are representative of other non-participating practices. While the QUALICOPC patient respondents are not representative of their physicians’ rosters or all Ontarians, their responses may still be representative of other patients with a similar health profile and possibly of patients who tend to visit their physicians.

With continued interest in primary care reform in Canada and throughout the world, the QUALICOPC study provides important data for further research. Several Canadian and international studies have already been published using the Canadian QUALICOPC data [32–36]. Our study describes the extent to which the Ontario QUALICOPC physician and patient respondents are representative of their practices and the general population, which is important for appropriately interpreting results of studies relying on Canadian QUALICOPC data. This study also highlights the importance of assessing nonresponse bias to appropriately generalize the results of surveys to certain populations. Knowledge of the characteristics of physicians and patients that are underrepresented in research may be helpful in considering survey recruitment and sampling strategies for future research, in order to maximize the representativeness of the sample [13].

### Limitations

This representativeness study has some limitations. The use of administrative databases allowed us to compare survey respondents with large cohorts of nonresponding physicians and patients. However, some characteristics relevant to this study were not available in health administrative databases, such as whether physicians are affiliated with academic institutions, or how many hours per week they work in their respective clinics. These characteristics may have impacted the actual survey responses of respondents and nonrespondents thus potentially contributing to nonresponse bias. We were also unable to identify and exclude primary care physicians who predominantly have a focused practice (e.g. sports medicine or travel medicine) from the comparison groups, even though they were not eligible to participate in the QUALICOPC.

We only examined the representativeness of the Ontario subset of the Canadian QUALICOPC respondents. Physician recruitment methods varied slightly between provinces; therefore, the generalizability of the physician component of this study is certain only in the province of Ontario [2]. The patients were recruited by similar consecutive visit-based sampling across the provinces. However, the generalizability of the patient results to the national sample depends on the extent to which the differences in physician sampling across provinces selected physicians with different patient populations. This study highlights a need to examine the representativeness of the QUALICOPC study in other Canadian jurisdictions, to appropriately contextualize the results of studies relying on Canadian QUALICOPC data.

## Conclusion

The physician respondents of the Ontario QUALICOPC differed slightly from their practice groups, and to a larger extent from other Ontario primary care physicians with respect to most of the characteristics studied. Visit-based sampling may have led to a biased patient respondent sample, in which the patient respondents of the Ontario QUALICOPC tended to be older, sicker, and more likely to be female than the other patient groups. However, despite these differences, Ontario QUALICOPC physician respondents had similar rosters overall compared to their practice groups and the general population.

These results will have implications for studies relying on QUALICOPC data as well as other primary care surveys. Those using QUALICOPC data should understand the limited representativeness of the respondents, and consider the potential for bias in their analyses. While physician and patient-level results are not representative of the entire Ontario population, the participating practices may be representative of other non-participating practices, and the patients selected by visit-based sampling may also be representative of visiting patients in other practices. Future primary care surveys are encouraged to consider consistent recruitment and sampling strategies across jurisdictions if possible, and to consider integrating measurement of nonresponse bias into survey protocols. We have demonstrated one method of assessing sample representativeness using administrative data, which could be used regardless of the sampling methodology selected.

## Additional file

Additional file 1: Primary Care Models. Summary of primary care models in Ontario including composition and characteristics, physician compensation type, and whether patient enrolment is required. (DOCX 14 kb)

## Abbreviations

CAPE: Client agency program enrolment tables
CHF: Congestive heart failure
CIHI-DAD: Canadian Institute of health information discharge abstract database
COPD: Chronic Obstructive pulmonary disease
ICES: Institute for clinical evaluative sciences
IPDB: ICES physician database
NACRS: National ambulatory care reporting system
OHIP: Ontario health insurance plan
QUALICOPC: Quality and costs of primary care study
RIO: Rurality index of ontario
RPDB: Registered person’s database
RUB: Resource utilization band
