# Supplementary material for: Assessing the representativeness of physician and patient respondents to a primary care survey using administrative data
Source: BMC Fam Pract. 2018 May 30;19:77. doi: 10.1186/s12875-018-0767-9 (PMC5977493; doi:10.1186/s12875-018-0767-9)
Supplement: Supplementary file 1 — Primary Care Models. Summary of primary care models in Ontario including composition and characteristics, physician compensation type, and whether patient enrolment is required. (DOCX 14 kb) [file 12875_2018_767_MOESM1_ESM.docx]

Additional file 1: Primary Care Models

| **Primary Care Delivery Model** | **Composition and Characteristics** | **Physician Compensation Model** | **Patient enrolment required (Rostering*)** |
| --- | --- | --- | --- |
| **Fee for Service (FFS)** | Solo physicians compensated for services performed according to the Schedule of Benefits. | Fee-for-service | No |
| **Comprehensive Care Model (CCM)** | Solo physicians providing comprehensive primary care services to enrolled patients and some after hours care. | Fee-for-service based plus after-hours premiums. Monthly capitation payments for enrolled patients. | Yes |
| **Family Health Group (FHG)** | Groups of physicians (3 or more) providing comprehensive care to enrolled patients on a 24/7 basis (through office hours and Telephone Health Advisory Services). | Fee for service plus after hours and comprehensive care premiums and bonuses, plus monthly comprehensive capitation payments. | Voluntary patient enrolment. |
| **Family Health Network (FHN)** | Groups of 3 or more physicians providing comprehensive care to patients through office hours and Telephone Health Advisory Service. **The main difference between FHN and FHO is the base rate payment and basket of core services.* | Capitated payments and fee-for-service. | Yes |
| **Family Health Organization (FHO)** | Groups of 3 or more physicians, providing care through regular and extended office hours, and nurse staffed Telephone Health Advisory Service.  **The main differences between FHN and FHO are the base rate payment and the basket of core services.* | Blended capitation model, with additional payments for delivering targeted care services. | Yes |
| **Family Health Team (FHT)** | Team of 3 or more physicians, with interdisciplinary health providers (nurses, social workers, dietitians, and others). | Physicians must be practicing within FHN or FHO, Blended capitation model, blended salary model, or complement-based remuneration plus bonuses and incentives. | Yes |
| **Other Model** | Other group models, including Community Health Group, Group Health Centre, and Rural and Northern Physician Group | Various | Various |

Blended payment: capitation, fee-for-service, premiums and bonuses.

*To roster, patients complete a Patient Enrolment and Consent to Release Personal Health Information form. Once this form is completed, the physician acknowledges it and sends it to the Ministry of Health and Long Term Care for processing. Patient enrolment is voluntary, however, physicians receive some incentives for enrolled patients only.

Sources: [37-39]
